# Mouthwash as a non-invasive method of indocyanine green delivery for near-infrared fluorescence dental imaging

**DOI:** 10.1117/1.JBO.27.6.066001

**Published:** 2022-06-10

**Authors:** Zhongqiang Li, Zheng Li, Waleed Zaid, Michelle L. Osborn, Yanping Li, Shaomian Yao, Jian Xu

**Affiliations:** a Louisiana State University, College of Engineering, Division of Electrical and Computer Engineering, Baton Rouge, Louisiana, United States; b Louisiana State University, School of Veterinary Medicine, Department of Comparative Biomedical Science, Baton Rouge, Louisiana, United States; c Louisiana State University Health Science Center, Oral and Maxillofacial Surgery, School of Dentistry, Baton Rouge, Louisiana, United States; d University of Saskatchewan, School of Environment and Sustainability, Saskatoon, Saskatchewan, Canada

**Keywords:** indocyanine green, near-infrared fluorescence dental imaging, oral administration, mouthwash, indocyanine green delivery

## Abstract

**Significance:**

X-ray imaging serves as the mainstream imaging in dentistry, but it involves risk of ionizing radiation.

**Aim:**

This study presents the feasibility of indocyanine green-assisted near-infrared fluorescence (ICG-NIRF) dental imaging with 785-nm NIR laser in the first (ICG-NIRF-I: 700 to 1000 nm) and second (ICG-NIRF-II: 1000 to 1700 nm) NIR wavelengths.

**Approach:**

Sprague Dawley rats with different postnatal days were used as animal models. ICG, as a fluorescence agent, was delivered to dental structures by subcutaneous injection (SC) and oral administration (OA).

**Results:**

For SC method, erupted and unerupted molars could be observed from ICG-NIRF images at a short imaging time (<1  min). ICG-NIRF-II could achieve a better image contrast in unerupted molars at 24 h after ICG injection. The OA could serve as a non-invasive method for ICG delivery; it could also cause the glow-in-dark effect in unerupted molars. For erupted molars, OA can be considered as mouthwash and exhibits outstanding performance for delivery of ICG dye; erupted molar structures could be observed at a short imaging time (<1  min) and low ICG dose (0.05  mg/kg).

**Conclusions:**

Overall, ICG-NIRF with mouthwash could perform *in-vivo* dental imaging in two NIR wavelengths at a short time and low ICG dose.

## 1 Introduction

To date, several imaging modalities have been commonly used for the diagnosis of dental diseases in dentistry, including x-ray imaging (e.g., panoramic radiography), ultrasound, and magnetic resonance imaging (MRI).^1^ Dental x-ray imaging is the most common imaging modality in the clinic.^1^^–^^4^ Cone-beam computed tomography (CBCT) provides more accurate diagnostic information about dental diseases by reconstructing three-dimensional dental structures related to dental diseases.^5^^,^^6^

However, the ionizing radiation from x-ray photons is a significant concern to the patient;^2^^–^^4^ for instance, if using CBCT for the examination, patients would receive 3 to 44 times higher ionizing-radiation dosage than the panoramic radiography [two-dimensional (2D) images].^5^ According to recent studies, ionizing radiation from x-ray imaging causes low-birth weight in babies and increases the incidence of cancer (especially thyroid cancer).^7^^,^^8^ Other imaging approaches, such as MRI, are limited by their high equipment or time cost, or their inability to accurately detect dental diseases.^4^^,^^9^^,^^10^

Optical imaging, especially fluorescence imaging, is becoming increasingly important in the biomedical sciences, because of its good imaging contrast, high sensitivity, and inexpensive cost.^11^^,^^12^ Many existing studies indicate that near-infrared (NIR) light looks more promising than visible light for the development of novel dental optical imaging techniques.^13^^–^^18^ As the only Food and Drug Administration (FDA)-approved NIR fluorescence dye for clinical usage in fluorescent angiography, indocyanine green has been widely used in many other fields, like cancer or dental imaging.^11^^,^^19^^–^^27^ Our previous studies have successfully demonstrated ICG-NIR fluorescence (ICG-NIRF) dental imaging in both human-extracted tooth and rat models.^17^^,^^28^^–^^32^

Regarding ICG-based imaging, most existing studies use ICG dye imaging in the short NIR window I (NIR-I, 700 to 1000 nm).^23^^,^^33^^–^^35^ However, long NIR windows (NIR-II: 1000 to 1700 nm) obtain a deeper tissue penetration and better image quality because of its low autofluorescence and photon scattering in biological tissues,^36^^,^^37^ and ICG could still yield a good spectral efficiency in the long NIR windows.^38^ In dentistry, many existing studies found that long NIR wavelengths exhibit a good efficiency in the detection of dental diseases.^17^^,^^28^^,^^39^^–^^44^ Several studies have successfully demonstrated that ICG can perform NIR-II imaging in thoracic malignancy,^45^ bile duct imaging,^46^ and human dental imaging.^17^^,^^47^ In our previous rat model studies, ICG-NIRF dental imaging was only performed in the short NIR wavelength.^29^^–^^32^ Few studies have reported using ICG for dental imaging in the second NIR windows to our best knowledge.

Meanwhile, in most of the existing studies related to *in-vivo* ICG imaging (cancer imaging or retinal angiography), ICG was administrated using invasive methods [e.g., tail-vein injection or subcutaneous injection (SC)].^11^ However, the invasive ICG injection for dental imaging may limit the wide usage of ICG-NIRF for dental imaging purposes.^31^ Mouthwash (or mouth rinse) is usually used for the maintenance of oral hygiene to decrease the chance of cavities and gum disease.^48^ For dental imaging, mouthwash is used to deliver gentian violet or methylene blue stains to enhance the imaging contrast of crack lesions for visual dental inspection.^49^ Our previous study had initially demonstrated the feasibility of ICG-NIRF dental imaging with mouthwash in NIRF-I (NIR-fluorescence-I: 700 to 1000 nm) but not in NIRF-II (NIR-fluorescence-II: 1000 to 1700 nm).^31^^,^^37^ Therefore, this study presents the feasibility of ICG-NIRF dental imaging in both NIRF-I and NIRF-II and includes optimizing conditions for ICG-NIRF dental imaging with the mouthwash method.

## 2 Methods and Materials

### 2.1 ICG-NIRF Dental Imaging System

In previous studies,^17^^,^^29^^–^^32^ we developed the ICG-NIRF dental imaging system for the rat model. It consisted of a 785-nm laser source (Turnkey Raman Lasers-785 Series; Ocean Optics, Inc), NIR-I cameras: Guppy F038B (CMOS sensor with 768×492 resolution) and Mako U-130B (CMOS sensor with 1280×1024 resolution) NIR cameras (Allied Vision Technologies GmbH) with 800-nm filter (long pass lens: 800 nm; Thorlabs Inc), QEPro spectrometer (Ocean Optics, Inc), and an OSF-3 endoscope (Olympus Corporation). In this study, to image rat molars in NIR-II, we further integrated InGaAs-based cameras in the ICG-NIRF imaging system: Goldeye G-008 (InGaAs FPA Sensor with 320×256 resolution, Allied Vision Technologies GmbH) and Goldeye G-033 NIR-II cameras (InGaAs FPA Sensor with 640×512 resolution, Allied Vision Technologies GmbH) with 1000-nm filter (long pass lens: 1000 nm; Thorlabs Inc).

### 2.2 Animals

The animal model was Sprague Dawley rats that were self-bred at the vivarium of the School of Veterinary Medicine, Louisiana State University. This project was approved by the Institutional Animal Care and Use Committee of Louisiana State University (Protocol No. 20-005) and all experiments were followed the ethical guidelines of animal care. ICG solution was prepared by dissolving ICG (Sigma-Aldrich, St. Louis, Missouri) in double-distilled water at the concentration of 1  mg/ml (about 1.29 mM).

In this study, a total of 92 rats with unerupted or erupted molars were used as shown in Tables 1 and 2. Among them, 36 P14 (postnatal 14 days) rats and 24 P21 (postnatal 21 days) rats were used to explore the efficiency of SC and OA methods for delivery of ICG using ICG-NIRF dental imaging in ICG-NIRF-I and ICG-NIRF-II (Table 1). The selection of P14 and P21 is mainly because of easier operation for SC and OA methods and observation of laser-treated molars with no effect of laser-treated injuries in unerupted molars (P14). The unerupted molars were imaged at <1  min, 10 min, 24 h, 48 h, and 72 h, when erupted molars were imaged at <1  min, 10 min, and 4 h (Table 1).

**Table 1:** Numbers of rats in SC and OA with low (1  mg/kg) and high ICG doses (5  mg/kg).

	Imaging time	SC		OA	
		Low dose	High dose	Low dose	High dose
Unerupted molars (P14)	<1 min	2	2	2	2
	10 min	2	2	2	2
	24 h	2	2	2	2
	48 h	2	2	2	2
	72 h	2	-	2	-
Erupted molars (P21)	<1 min	2	2	2	2
	10 min	2	2	2	2
	4 h	2	2	2	2

**Table 2:** Numbers of rats used for the OA ICG delivery with different ICG doses and the laser treatment with OA.

Different ICG doses with OA		Laser treatment with OA	
ICG doses (mg/kg)	P60 rats	Postnatal days	Rats
0.05	2	P14	6
0.1	2	P21	14
0.5	2	P60	4
1	2	—	—

Another 8 P60 (postnatal 60 days) rats were used to investigate the influences of ICG doses with OA (Table 2). To explore different ICG doses with OA delivery, by using a pipette, P60 rats were orally administrated (ICG concentration: 1  mg/ml) at different ICG doses: 0.05, 0.1, 0.5, and 1  mg/kg; two P60 rats were used for each dose (Table 2).

We also treated molars with the laser to induce abnormal eruption. For the laser treatment, there were a total of 24 rats that were laser-treated left molars at P9 (laser power: 0.8 W and treatment time: 10 s, laser density: about $101\;W/{\mathrm{cm}}^{2}$ with the laser spot 1 mm in diameter); to monitor the eruption of the laser-treated molars, six of the 24 rats were sacrificed at P14 and image after 10 min of OA, 14 of them were imaged at P21, while four of them were imaged at P60 (Table 2). All those rats were orally administrated with 10-μl ICG solutions and were imaged after 10 min of ICG OA treatment.

After the denigrated imaging time, the rats were euthanized by isoflurane anesthesia, followed by cervical dislocation; then, rat mandibles were extracted and imaged under the ICG-NIRF imaging system.

### 2.3 Image Contrast

In our previous study,^32^ we defined an image contrast to quantitatively evaluate the imaging quality of ICG-NIRF dental imaging the under different conditions, and it could be calculated through Eq. (1), which cold efficiently fluorescence difference between molar regions and the surrounding tissues (background):

$${\text{Image}}_{\text{constrast}}=\mathrm{avg}(\overset{n}{\underset{i=0}{\sum }}\frac{\left(p_{m}-p_{b}\right)}{\mathrm{avg}(\sum_{i=0}^{n}p_{m})+avg(\sum_{i=0}^{n}p_{b})}),\;$$ (1)

where $p_{m}$ and $p_{b}$ stands for the pixel values from the molar regions and the background, respectively, when n is the number of the pixels in the sampling lines. The ranges of image contrast are [0, 1]: the contrast towards to 0 means that molar almost has no difference to the background tissues, while the contrast closer to 1 means that the difference of molar and background is larger.

### 2.4 Statistical Analysis

SPSS 28 software (IBM Inc.) was used to perform statistical analysis. One-way Analysis of variance and paired t-test were used to analyze among different treatments. The p values of <0.05 (*), <0.01 (**), and <0.001 (***) were statistically significant.

## 3 Results

### 3.1 ICG Delivery by Using SC and OA Methods for ICG-NIRF Dental Imaging

**Fig. 1:**
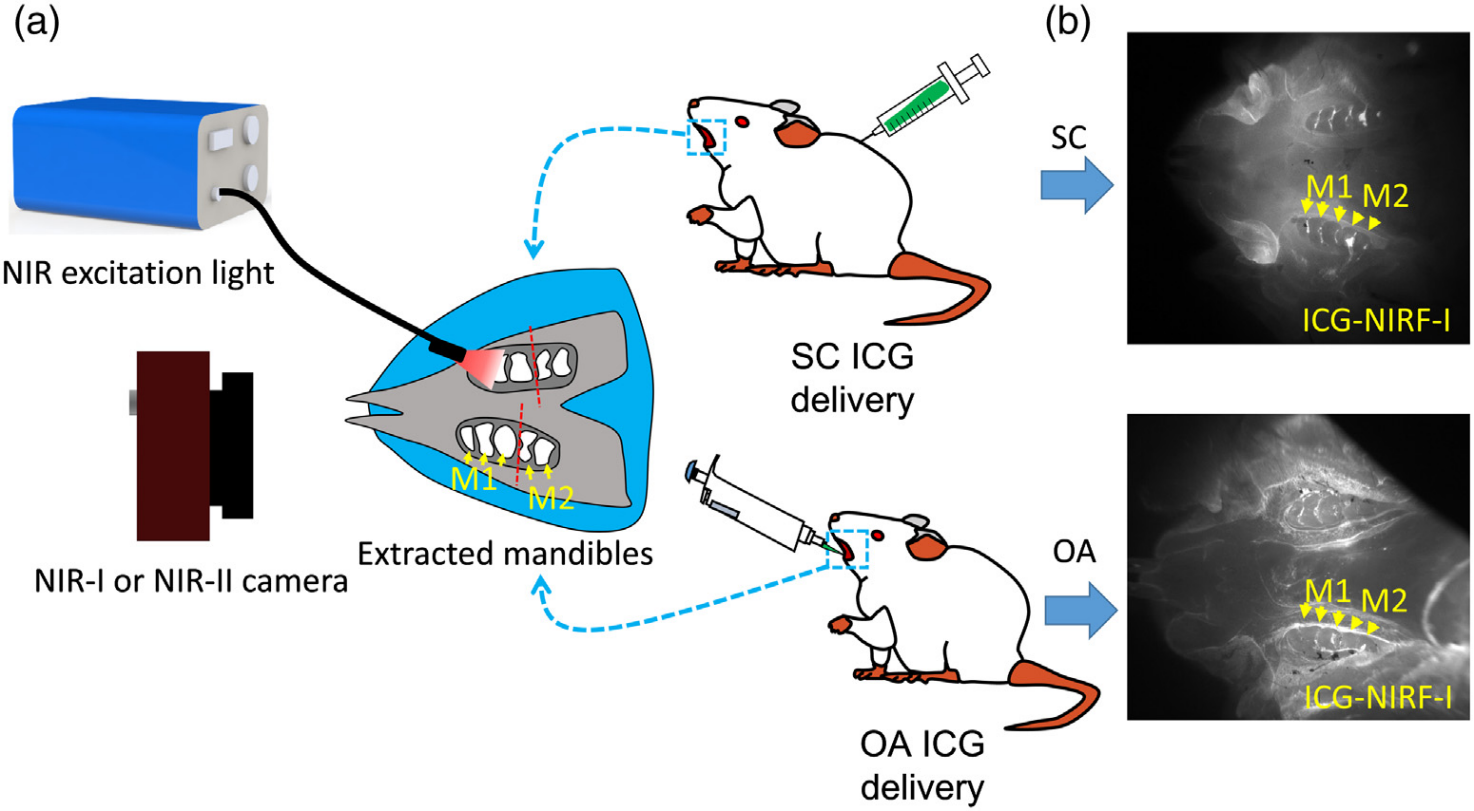
Schematic diagram of ICG-NIRF dental imaging with SC and OA ICG delivery. (a) Schematic diagram and (b) typical ICG-NIRF images of P21 erupted molars with SC (4 h) and OA (10 min) methods. M1: first molar; M2: second molar.

Figure 1(a) shows the schematic diagram of the ICG-NIRF dental imaging with SC and OA ICG delivery. For the SC method, the specific volume of ICG dye was administrated into the rats from the dorsum. For the OA, a pipette was used to measure the same amount of ICG dye, and then the ICG dye was orally administered. The rats were sacrificed at the designated time points, and the mandible was extracted and placed under NIR-I or NIR-II camera to perform ICG-NIRF dental imaging [Fig. 1(a)].

In the OA method, ICG agents were delivered directly to the erupted molars, and ICG-NIRF dental imaging could almost immediately perform at the short imaging time.^31^ The OA method could also be considered as the mouthwash for the erupted molar at the short imaging time. Figure 1(b) indicates the typical ICG-NIRF-I images of P21 erupted molars with SC injection (imaging time: 4 h) and OA (imaging time: 10 min), through which the three cusps of the first molars could be observed and recognized.

### 3.2 SC-Based ICG-NIRF Dental Imaging in NIRF-I and NIRF-II

**Fig. 2:**
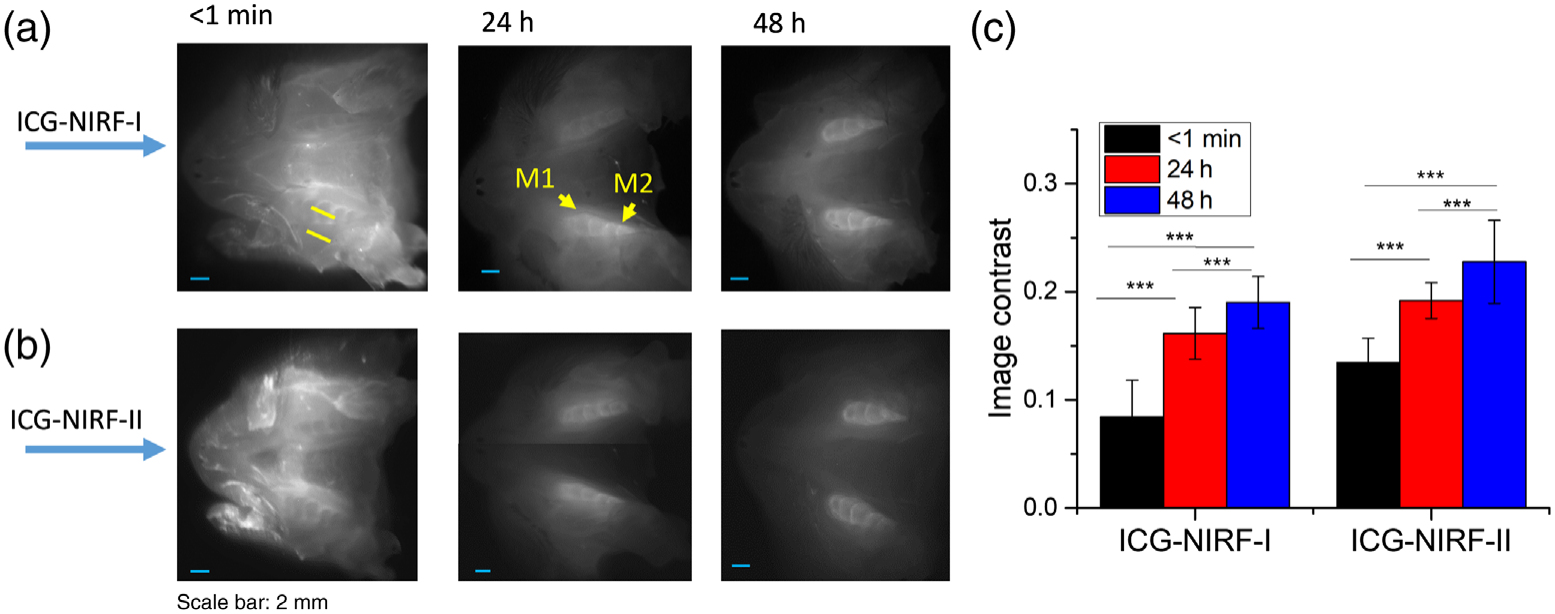
The SC-based ICG-NIRF imaging for unerupted molars under the first (ICG-NIRF-I) and second (ICG-NIRF-II) NIR windows. (a) ICG-NIRF-I; (b) ICG-NIRF-II; and (c) image contrast of ICG-NIRF-I and ICG-NIRF-II under different imaging times. M1: the first molar; M2: the second molar. ICG dose: 5  mg/kg. * is significant at <0.05; ** is significant at <0.01; and *** is significant at <0.001.

Figure 2 shows ICG-NIRF-I and ICG-NIRF-II imaging P14 unerupted molars under different imaging times (<1  min, 24 h, and 48 h). The SC-based ICG-NIRF dental imaging could recognize the first molars at a short imaging time (<1  min). The molar regions in ICG-NIRF-I and ICG-NIRF-II were relatively darker than the surrounding tissues. The glow-in-dark effect could be observed under both NIR windows after 24 h of ICG injection, where the molar regions remained bright while the surrounding tissues became dark.

Compared to 24 h, 48 h had a clearer molar profile that the three cusps of the first molars could be seen under both ICG-NIRF-I and ICG-NIRF-II. From the image contrast [Fig. 2(c)], ICG-NIRF-II had a relatively larger image contrast than ICG-NIRF-II; longer imaging time (within 48 h) had a better imaging quality than the imaging time within 24 h.

**Fig. 3:**
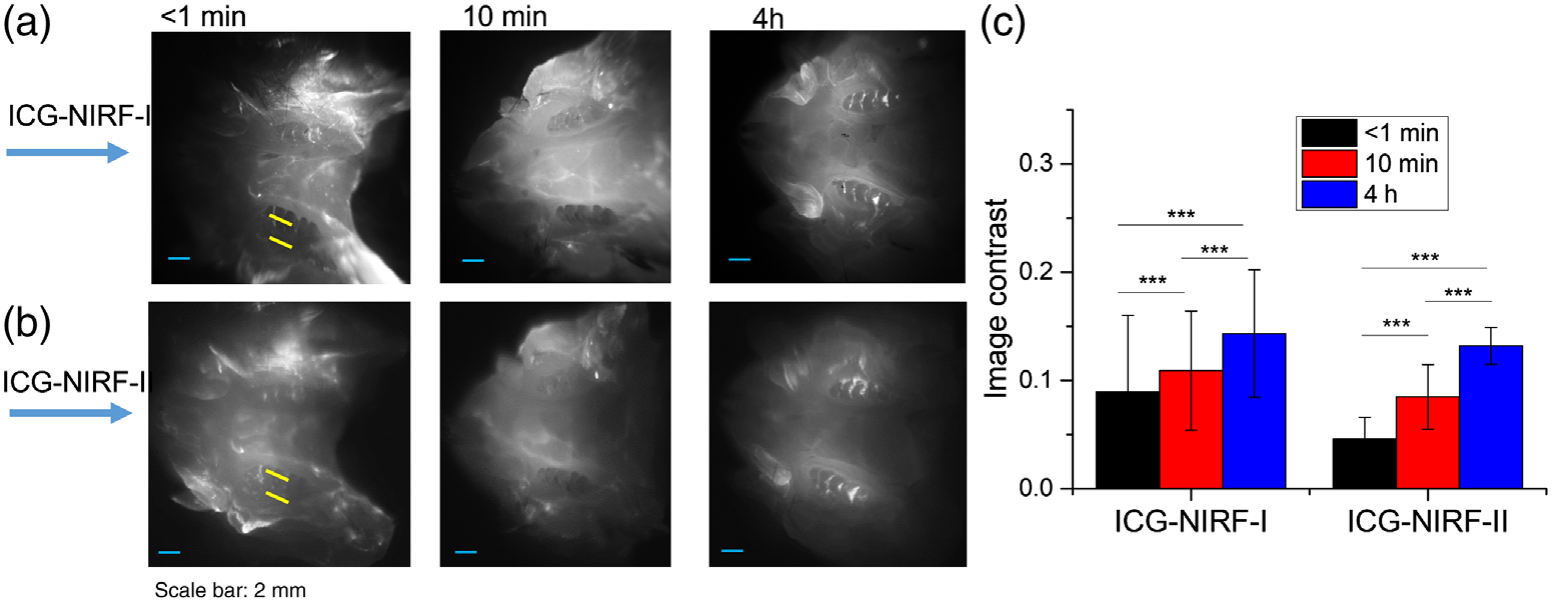
The SI-based ICG-NIRF dental imaging of the erupted molars (P21) under NIRF-I and NIRF-II. (a) ICG-NIRF-I; (b) ICG-NIRF-II; and (c) image contrast of ICG-NIRF-I and ICG-NIRF-II under different imaging times. ICG dose: 5  mg/kg. * is significant at <0.05; ** is significant at <0.01; and *** is significant at <0.001.

For the erupted molars (P21), ICG-NIRF could acquire dental imaging under both NIRF-I and NIRF-II (Fig. 3). ICG-NIRF-I could immediately obtain a more explicit molar profile at a short imaging time (<1  min), but the molar became kind of blurred under ICG-NIRF-II. With increasing the imaging time (10 min and 4 h), the first molars were becoming much clearer under ICG-NIRF-I. After 4 h of ICG injections, the first molars could be observed under both ICG-NIRF-I and ICG-NIRF-II. The image contrasts could further confirm that the contrast increased with a long time after ICG injection [Fig. 3(c)]. Overall, ICG-NIRF-I has a better imaging quality than ICG-NIRF-II in the erupted molars.

### 3.3 OA-Based ICG-NIRF Dental Imaging with Oral Administration for the Unerupted and Erupted Molars

**Fig. 4:**
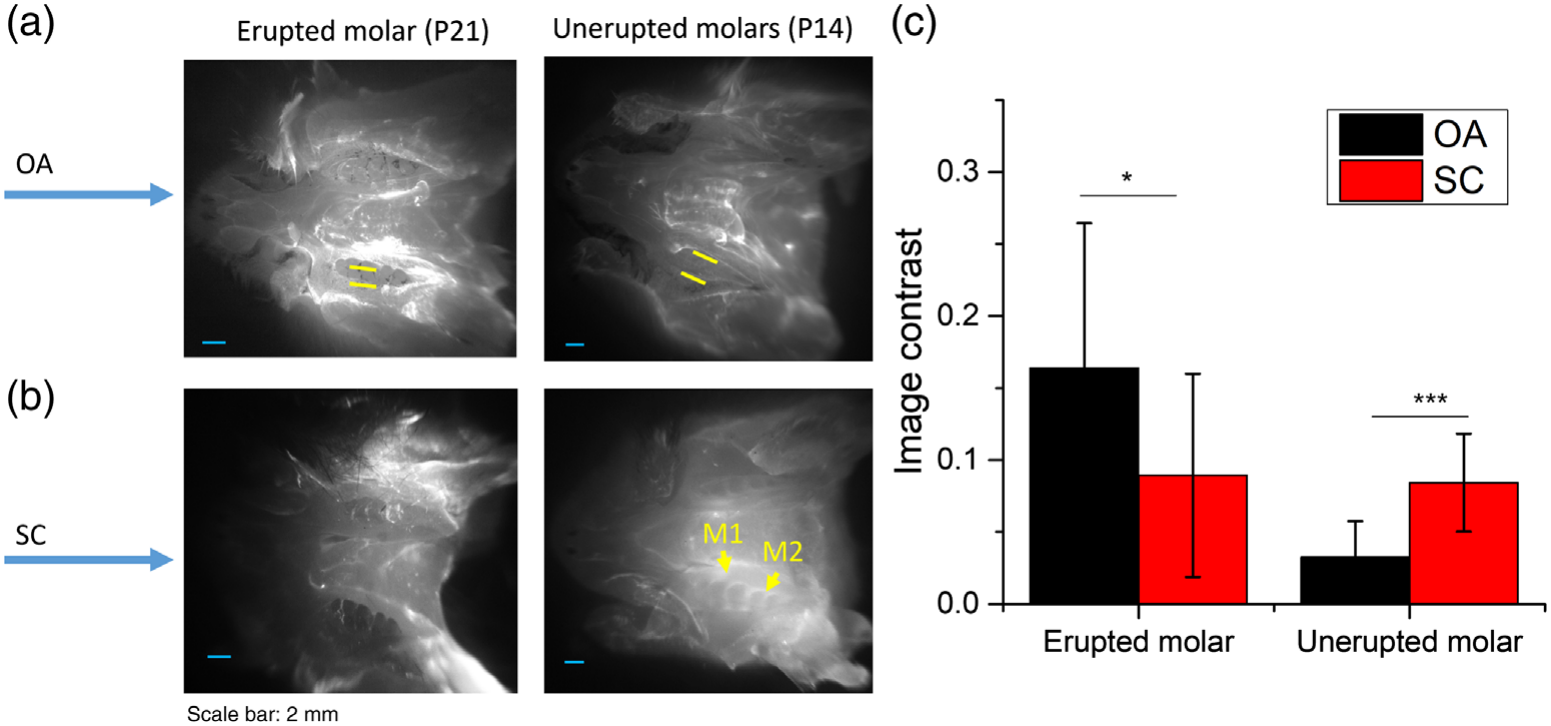
Comparison of OA and SC ICG delivery, erupted and unerupted molars were imaged at short imaging time (<1  min) with ICG-NIRF-I imaging. (a) Erupted and unerupted molars by using OA ICG delivery. (b) Erupted and unerupted molars by using SC ICG delivery; (c) Image contrast of OA and SC method. M1: the first molar; M2: the second molar. ICG dose: 5  mg/kg. * is significant at <0.05; ** is significant at <0.01; and *** is significant at <0.001.

Figure 4 shows the comparison of unerupted molars and erupted molars by using OA and SC ICG delivery. For the erupted molars, the first molars could be seen immediately with both ICG delivery methods under ICG-NIRF-I. Compared to the SC method, OA could obtain a more precise molar profile, which OA had a larger image contrast than the SC method [Fig. 4(c)]. For the unerupted molars, no dental structure could be observed for the OA method under short imaging time (<1  min), but the first molars and part of the second molars could be recognized from ICG-NIRF-I images with SC methods [Fig. 4(b)]. The image contrast of the SC method was also much larger than the OA method [Fig. 4(c)].

**Fig. 5:**
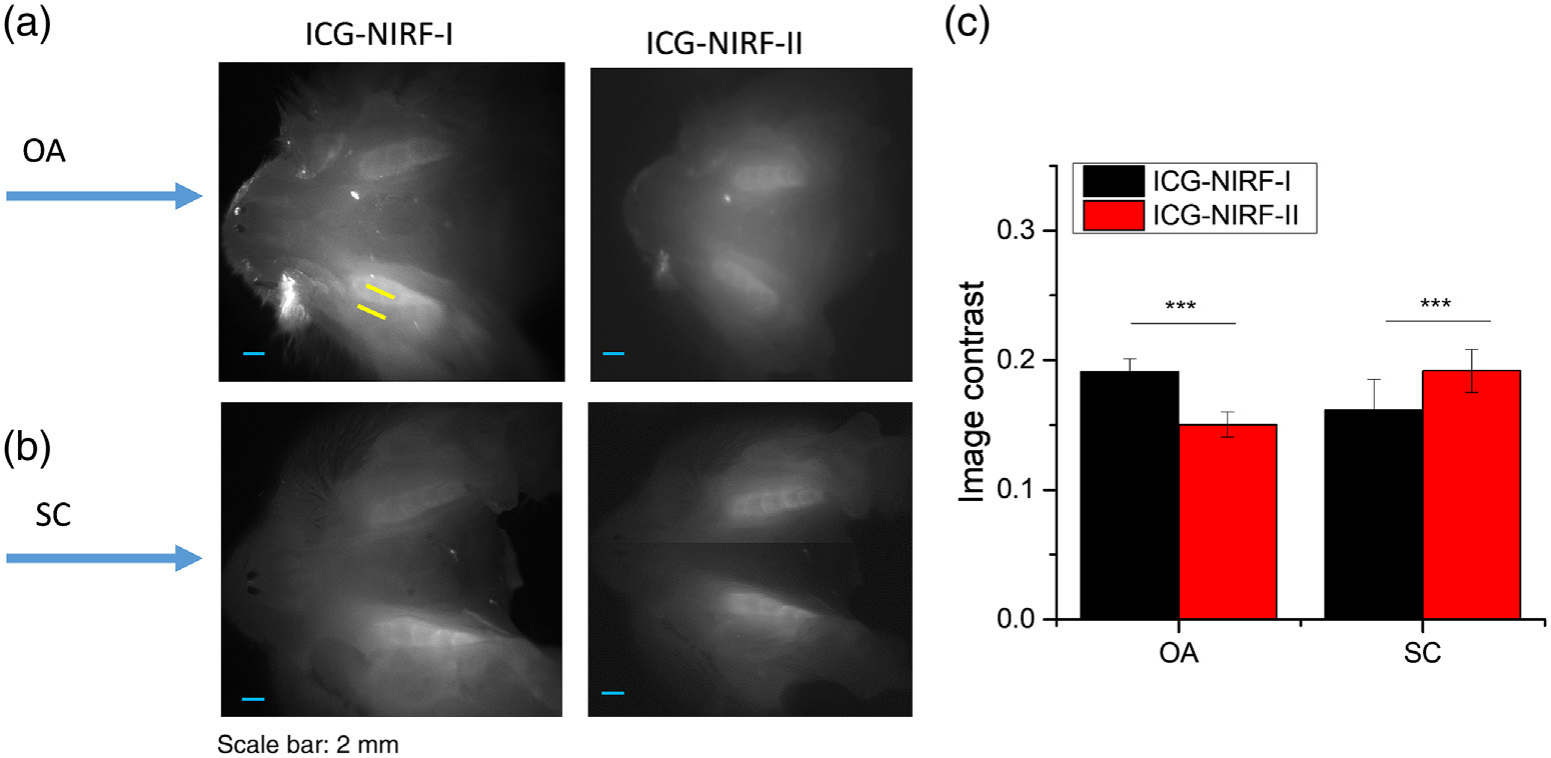
Comparison of OA-based and SC-based ICG-NIRF dental imaging in the unerupted molars at long imaging time (24 h). (a) The ICG-NIRF dental imaging with OA ICG delivery; (b) ICG-NIRF dental imaging with SC ICG delivery; and (c) image contrast of OA and SC methods. M1: the first molar; M2: the second molar. ICG dose: 5  mg/kg. * is significant at <0.05; ** is significant at <0.01; and *** is significant at <0.001.

However, in the unerupted molars, the glow-in-dark effect could also be observed under the OA method. From Fig. 5, it could be seen that the molar regions with the OA method became much brighter than the surrounding tissues after 24 h, which was similar to the SC method [Fig. 5(b)]. Comparing the ICG-NIRF-I and ICG-NIRF-II, the OA method could achieve a better imaging quality (larger image contrast) in ICG-NIRF-I than that in ICG-NIRF-II; whereas a better image contrast could be found in SC-based ICG-NIRF-II.

**Fig. 6:**
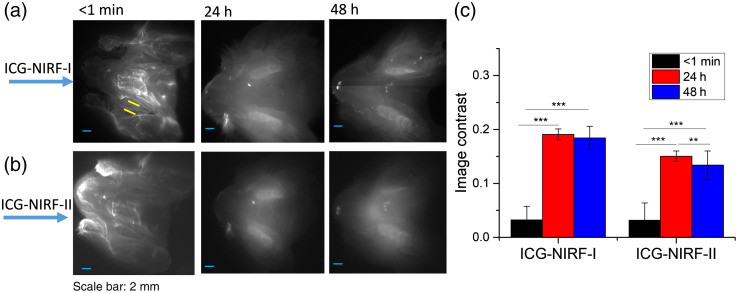
The OA-based ICG-NIRF imaging for unerupted molars under the first (ICG-NIRF-I) and second (ICG-NIRF-II) NIR windows. (a) ICG-NIRF-I; (b) ICG-NIRF-II; and (c) image contrast of ICG-NIRF-I and ICG-NIRF-II under different imaging times. * is significant at <0.05; ** is significant at <0.01; and *** is significant at <0.001.

In the unerupted molars, the OA method had inferior imaging quality at the short imaging time (<1  min), and no dental structures (or molar cusps) could be identified from ICG-NIRF-I or ICG-NIRF-II images (Fig. 6). After 24 h of ICG delivery, with only ICG molecules trapped in the molar regions, the glow-in-dark effect could greatly help to improve the imaging quality, and each molar crown could be observed from ICG-NIRF-I images. Compared to ICG-NIRF-I, there was a slight decrease in the image contrast for ICG-NIRF-II, and image contrast with 24 h of ICG delivery was slightly larger than that of 48 h in both ICG-NIRF-I and ICG-NIRF-II [Fig. 6(c)].

**Fig. 7:**
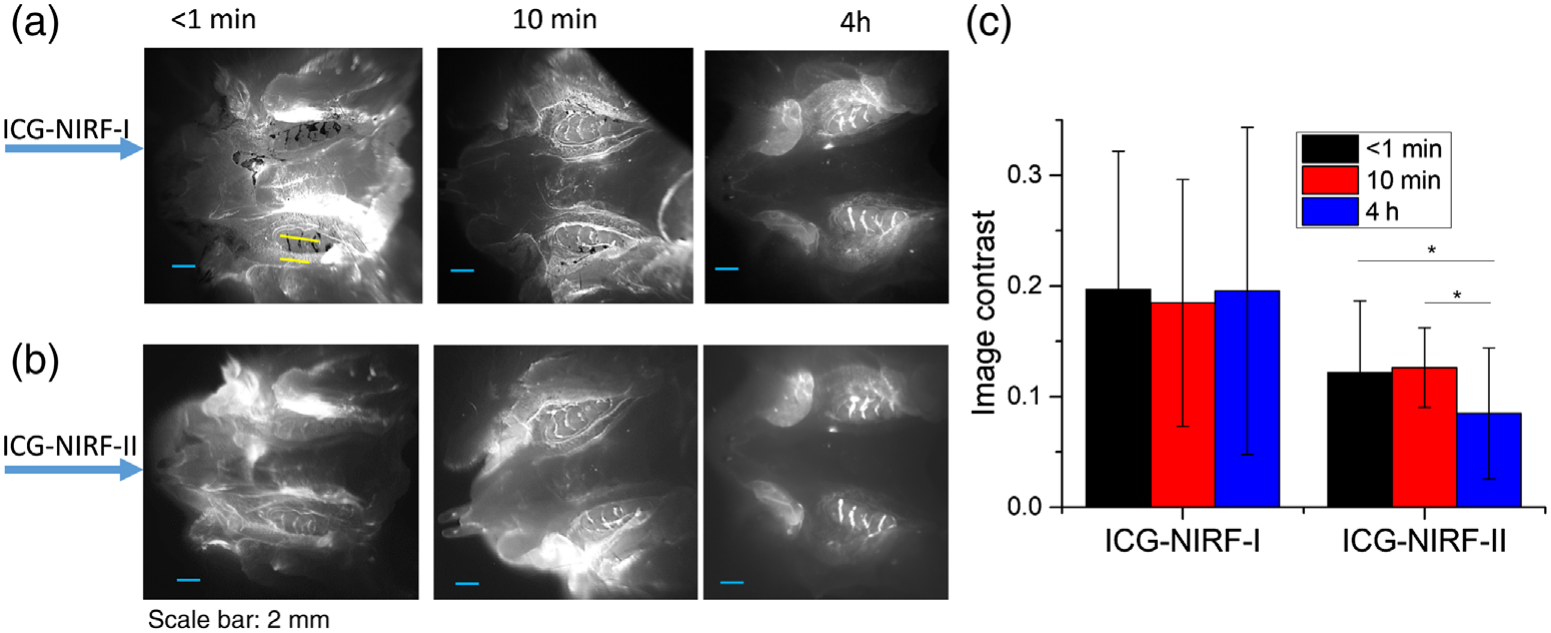
The OA-based ICG-NIRF dental imaging for the erupted molars (P21) under NIRF-I and NIRF-II. (a) ICG-NIRF-I; (b) ICG-NIRF-II; and (c) image contrast of ICG-NIRF-I and ICG-NIRF-II under different imaging times. * is significant at <0.05; ** is significant at <0.01; and *** is significant at <0.001.

For the erupted molar, molar regions could be seen from ICG-NIRF-I images immediately after OA, but they became a little blurred under ICG-NIRF-II images (Fig. 7). With a longer time (10 min) of ICG delivery, the molars were becoming slightly clearer, especially three cusps of the first molars that could be easily identified from ICG-NIRF-I images (Fig. 7). When increasing to 4 h of ICG delivery, the surrounding tissues became darker, while the molars still could be observed from both ICG-NIRF-I and ICG-NIRF-II images [Fig. 7(c)].

### 3.4 Effect of ICG Dose for OA-Based ICG-NIRF Dental Imaging

**Fig. 8:**
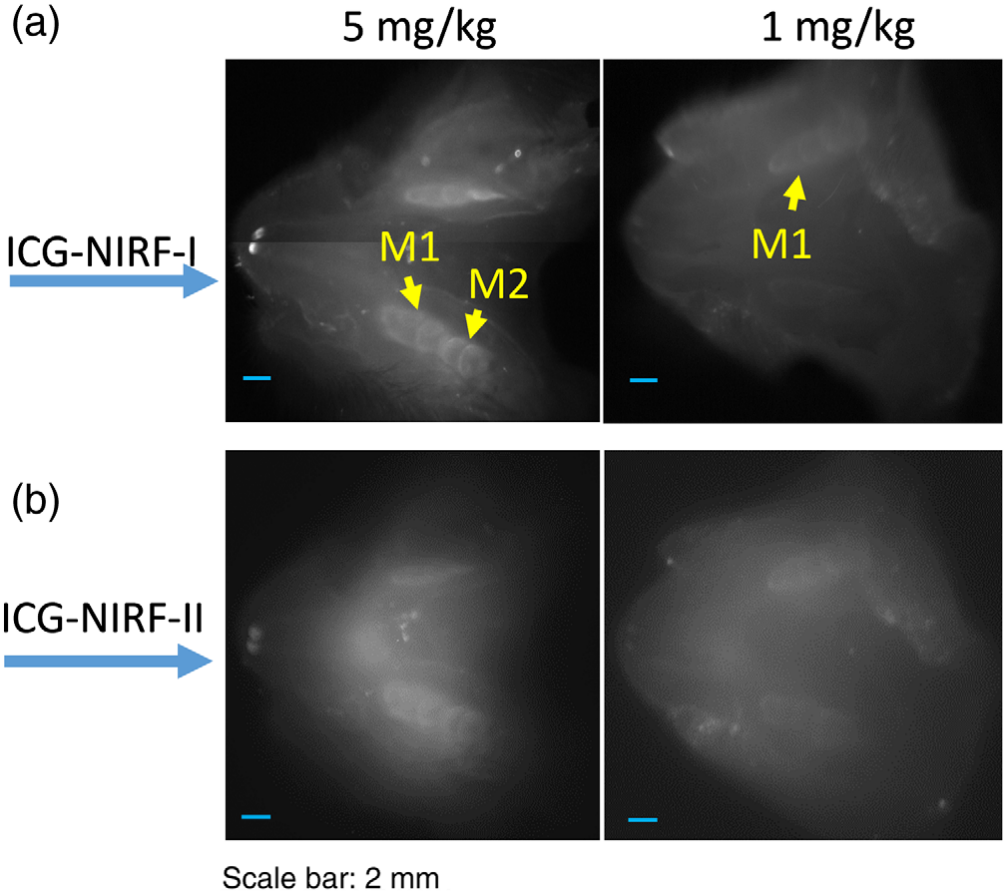
The OA-based ICG-NIRF dental imaging of the unerupted molars (P14) with low (1  mg/kg) and high (5  mg/kg) ICG doses, and imaging after 48 h of the OA ICG delivery. (a) ICG-NIRF-I dental imaging with low and high ICG and (b) ICG-NIRF-II dental imaging with low and high ICG.

We investigated the effect of ICG dose on OA-based ICG-NIRF dental imaging. Figure 8 indicates ICG-NIRF images of P14 rats with 48 h of OA method under low (1  mg/kg) and high (5  mg/kg) ICG dose. At a high ICG dose (5  mg/kg), both the first and second molars could be observed under ICG-NIRF-I and ICG-NIRF-II imaging. However, if the unerupted molars with low ICG dose (1  mg/kg), the glow-in-dark effect of the unerupted molars became not as clear as the high ICG dose; only the right-side first molars could be seen from ICG-NIRF-I images, but dental structures were blurred from ICG-NIRF-II images.

**Fig. 9:**
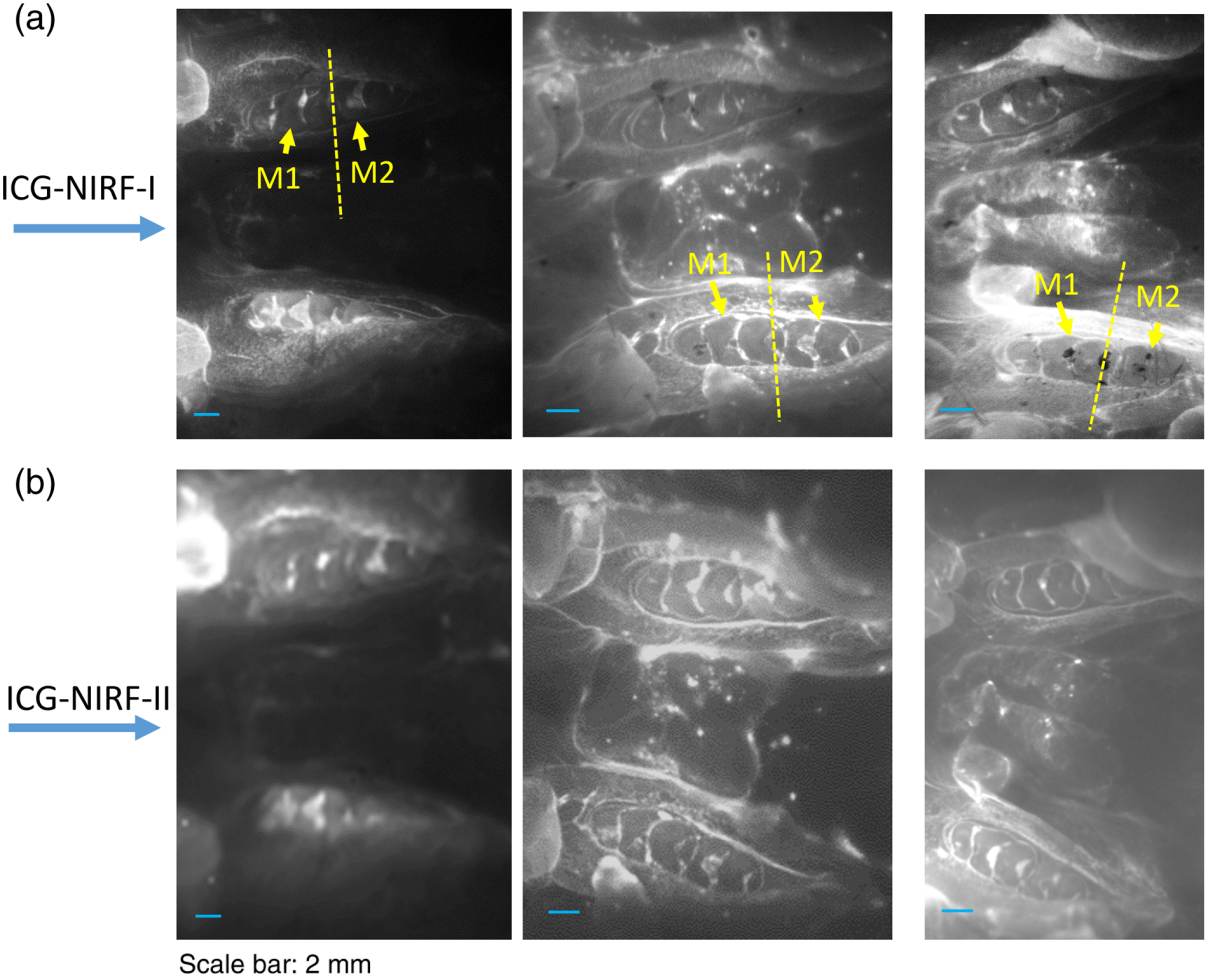
The OA-based ICG-NIRF dental imaging of the erupted molars (P60 young adult rats) with ICG dose ranging from 0.05 to 1  mg/kg, and imaging under NIRF-I and NIRF-II. (a) ICG-NIRF-I dental imaging and (b) ICG-NIRF-II dental imaging.

In the P60 adult rats, OA-based ICG-NIRF dental imaging could obtain clear profiles of both first and second molars at a low ICG dose (only 0.05  mg/kg), which OA could directly deliver ICG dye into molar regions. Both ICG-NIRF-I and ICG-NIRF-II could have a good imaging quality under ICG doses ranging from 0.05 to 1  mg/kg (Fig. 9). Especially, the three cusps of the first molars (M1) and two cusps of the second molars (M2) could be recognized from both ICG-NIRF-I and ICG-NIRF-II images. This means that ICG dose almost did not affect the OA-based ICG-NIRF dental imaging

### 3.5 Detection of Laser-Treated Molars by OA-ICG-NIRF Dental Imaging

**Fig. 10:**
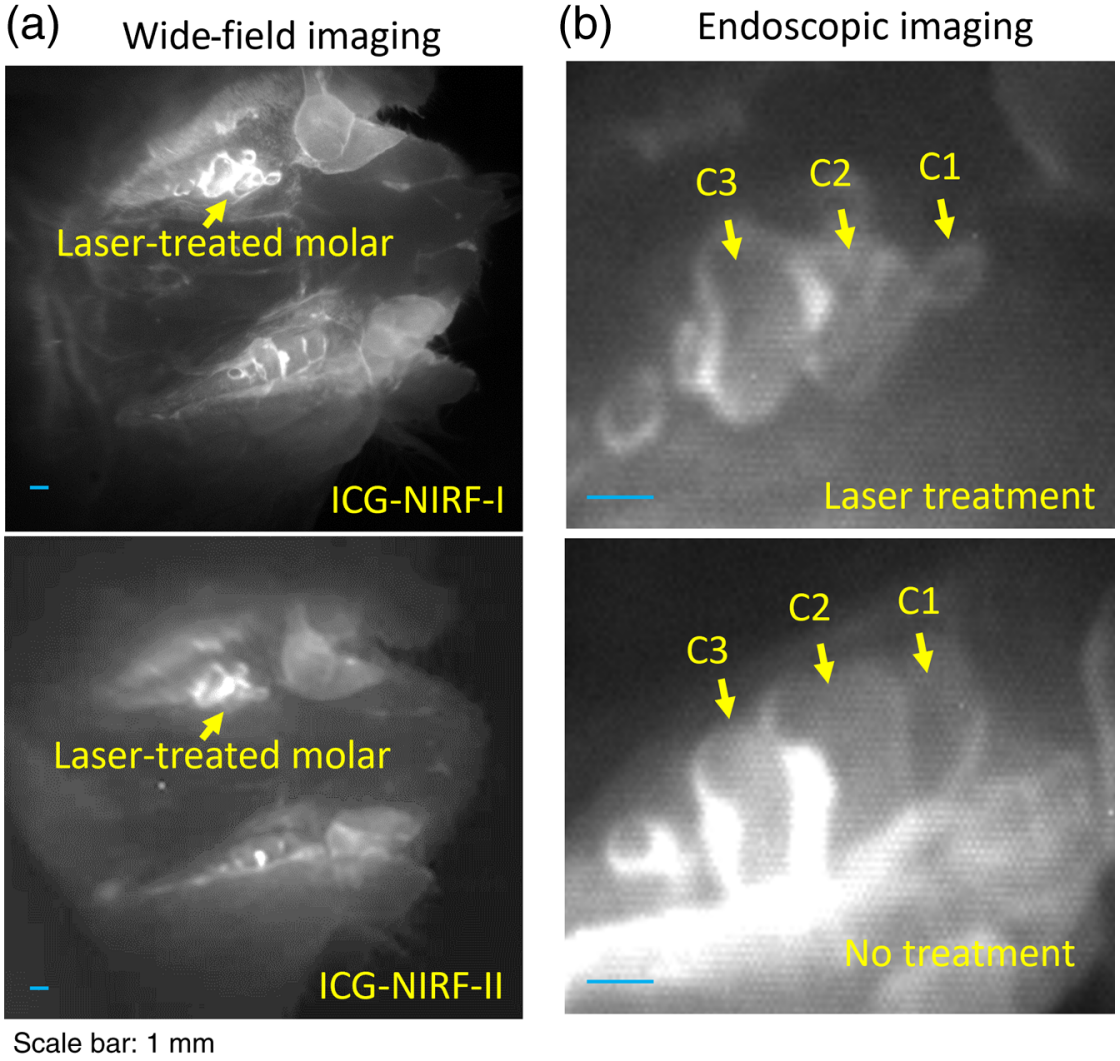
The OA-based ICG-NIRF imaging for the laser-treated abnormal erupted molars in P21 rats. (a) Wide-field pictures of abnormal erupted molar. (b) The endoscopic ICG-NIRF images of abnormal erupted molar and normal molar. C1: the first cusps; C2: the second cusps; and C2: the third cusps.

For the laser-treated molars, the OA method could have a good efficiency for ICG-NIRF dental imaging to recognize the molars of abnormal eruption in both ICG-NIRF-I and ICG-NIRF-II (Fig. 10). From the wide-field imaging, three cusps of un-treated first molar could be observed from ICG-NIRF-I images. The laser-treated molar abnormally erupted, especially only a small part of the first cusp towards to lingual side erupted. Under ICG-NIRF dental imaging, the laser-treated molars were brighter when compared to the un-treated molars. From the endoscopic dental imaging [Fig. 10(b)], the profiles of laser-treated molars could be greatly improved; compared to the un-treated molars, the first two cusps of the laser-treated molar only erupted partially.

**Fig. 11:**
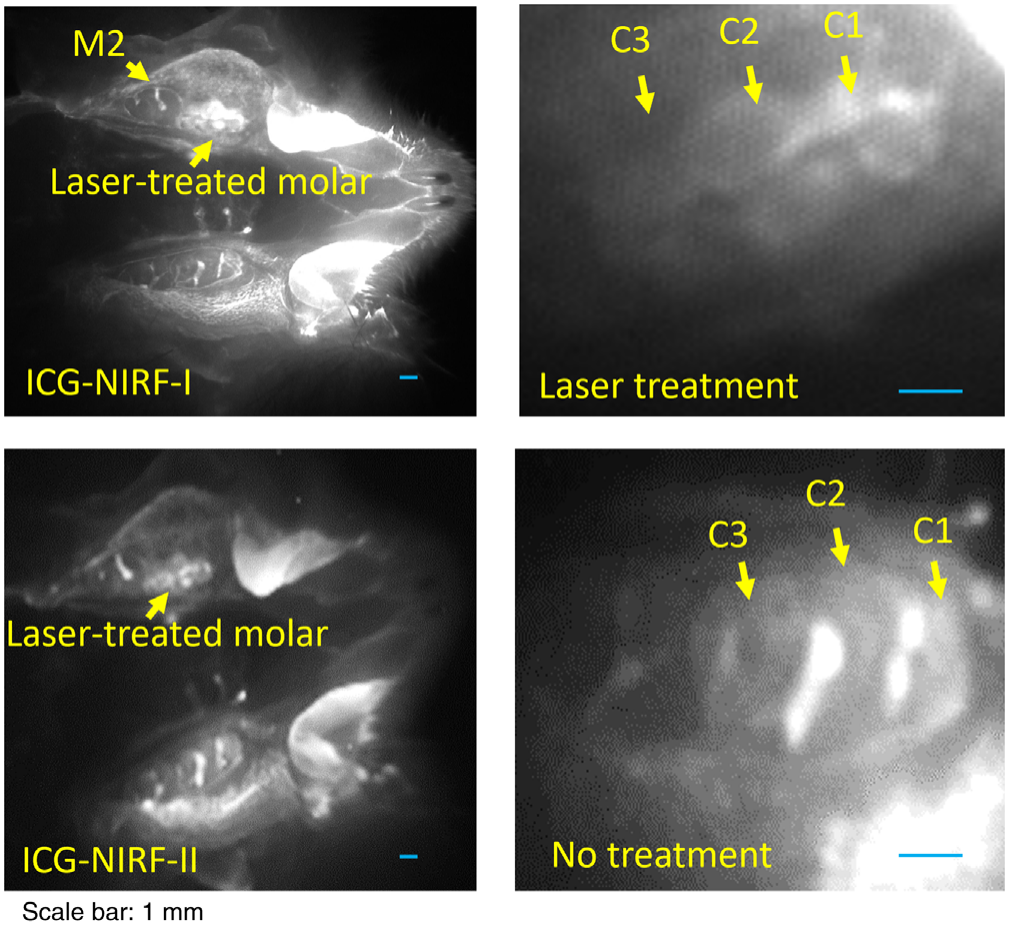
The OA-based ICG-NIRF imaging for the laser-treated abnormal erupted molars in young adult (P60) rats. (a) Wide-field pictures of abnormal erupted molar. (b) The endoscopic ICG-NIRF images of abnormal erupted molar and normal molar. C1: the first cusp; C2: the second cusp; and C2: the third cusp.

For the young adult (P60) rats, the laser-treated molars still erupted partially after laser treatment of dental follicles; these molars could be easily recognized by ICG-NIRF dental imaging, especially when compared to the completely erupted second-molars (M2). From ICG-NIRF-I, similar to P21 rats in Fig. 10, the laser-treated molar had a relatively stronger brightness than the un-treated molars. From the endoscopic imaging, compared to the un-treated molars, the entire laser-treated first molar incompletely erupted, especially only partially erupted on the lingual side of the first cusp (C1) [Fig. 11(b)].

## 4 Discussion

In ICG-based imaging, most of the existing studies have performed dental imaging in the NIRF-I (700 to 1000 nm).^23^^,^^33^^–^^35^ Compared with traditional NIRF-I, imaging with NIRF-II (1000 to 1700 nm) obtained deeper tissue penetration depth. It acquired good imaging quality because of its low background noises from tissue autofluorescence and photon scattering.^36^ Studies have demonstrated the successful use of ICG to perform NIRF-II imaging to detect thoracic malignancy^45^ and for imaging bile duct.^46^ Few studies have reported using ICG for ICG-NIRF dental imaging in NIRF-II in rat models to the best of our knowledge. This study successfully demonstrated that ICG-NIRF dental imaging could perform imaging in both first and second NIR windows in the rat models. In particular, the ICG-NIRF-II could even obtain a better imaging quality than ICG-NIRF-I of unerupted molars with the SC method due to the low background noise and excellent tissue penetration in NIR-II region. In addition, our previous study in human dentistry also demonstrated that some dental tissues, e.g., enamel, became transparent in NIR-II region, which could obtain a higher efficiency in the detection of dental diseases.^28^

In our previous study,^31^ we first reported the feasibility of oral administration (we also named mouthwash) to perform ICG-NIRF dental imaging using the living animal. The results show that OA could serve as a new non-invasive method for ICG delivery. But there are some limitations in our study: ICG-NIRF dental imaging was only performed in the NIRF-I, not in NIRF-II; only short imaging time (10 min) and low dose were investigated; OA or mouthwash was not well distinguished and defined.

In this study, we systematically explore more parameters that could help to improve the image quality of OA-based ICG-NIRF dental imaging. For the unerupted molars with the OA method, the glow-in-dark effect was observed at 24 h of ICG delivery, and both ICG-NIRF-I and ICG-NIRF-II could obtain a good image contrast at a long imaging time (24 to 48 h). However, no dental structures of the unerupted molars were observed at the short imaging time (<1  min), when the SC method could obtain clear molar profiles. In addition, ICG dose has a potential effect on OA-based ICG-NIRF dental imaging, which high ICG dose could achieve a good image quality with the glow-in-dark effect, but poor imaging quality was found at the low ICG dose (1  mg/kg).

For ICG-based medical imaging, oral administration of ICG dye is not commonly used,^50^ because if ICG is administered orally, it would be absorbed in the digestive system, and would cost about 1 h to reach the systemic circulation in the mouse model.^50^ Most ICG dye would access the hepatic portal system and be carried to the liver by portal vein,^51^ and the liver would extract over 70% of ICG dye in healthy humans.^52^^,^^53^ Therefore, a high ICG dose with the OA method would have more ICG dye absorbed by the digestive system, and more ICG dye could enter the systemic circulation and reach dental tissues. This is why a high ICG dose could achieve a better imaging quality than the low ICG dose in the unerupted molars.

For the erupted molars, the erupted molars tend to have a better imaging quality at the short imaging time, and ICG-NIRF-I had a better image contrast than ICG-NIRF-II. For the ICG dose, it was found almost no effect on the imaging quality: OA could still obtain clear molar profiles, and each cusp of both first and second molars were identified at a low ICG dose (0.05  mg/kg), and this dose was much lower than the existing studies related to the cancer imaging (usually larger than 1  mg/kg).^54^^–^^56^

From the results of unerupted and erupted molars, at the short imaging time (<1  min), no profiles of the unerupted molars could be recognized from ICG-NIRF images, but the erupted molars could be clearly observed and be almost not affected by ICG dose. Thus, at the short imaging time (e.g., <1  min), OA could be considered as the mouthwash because ICG dye could be delivered to the erupted molars directly and could be immediately performed ICG-NIRF imaging. The principle is like our previous studies using human extracted teeth, in which the extracted teeth were immersed into ICG solution and could be immediately profiled at a short imaging time (<1  min).^17^^,^^28^^,^^31^ But in the unerupted molars, the condition is a little different from the erupted molars: with increasing the imaging time, ICG molecules (orally administrated) would be absorbed in the digestive system at the beginning then would be delivered and trapped in the dental tissues. Eventually, the glow-in-dark effect could be observed in ICG-NIRF imaging.^30^^,^^32^ Therefore, OA could be considered as the mouthwash for the erupted molars at the short imaging time; OA could serve as a non-invasive and patient-friendly approach of ICG delivery for dental imaging with low ICG dose, and it could deliver ICG dye to the dental structures more efficiently.

In addition, the dental follicle plays an essential role in the tooth eruption. In this study, the laser delivers high energy in the form of light to the tissue; the laser vaporizes the dental follicle tissues that are in contact with laser; then, the defects or injuries in the dental follicle would lead to the abnormal eruption of the molar.^57^ Unerupted or impacted wisdom teeth are one of the most common dental diseases that affects about 25% to 50% of the population^6^^,^^58^^,^^59^), and early surgical removal could greatly reduce the risks of inferior alveolar nerve) injury and bone defects.^60^ Our previous study indicates that ICG-NIRF dental imaging could detect not only naturally abnormal erupted molars but also laser-treated molars.^29^^,^^32^ In this study, ICG-NIRF dental imaging could identify the laser-treated molars in both ICG-NIRF-I and ICG-NIRF-II imaging; mouthwash also efficiently delivered ICG dye to the laser-treated molars and facilitated the shorter dental imaging time (<1  min).

## 5 Conclusions

This study successfully demonstrated that ICG-NIRF dental imaging could be performed in both NIRF-I and NIRF-II in the rat models. For the SC method, the erupted molars and unerupted molars could be observed from ICG-NIRF images at a short imaging time (<1  min), but the unerupted molars have much longer imaging time than the erupted molars. ICG-NIRF-II could have a better image contrast than ICG-NIRF-I imaging after 24 h of ICG injection. OA could serve as a novel non-invasive method for the ICG delivery of ICG-NIRF dental imaging. It could also cause the glow-in-dark effect in the unerupted molars, which greatly helps to improve the imaging quality for the unerupted molars. For the erupted molars, OA could be considered as the mouthwash, and it shows the outstanding performance to efficiently deliver ICG dye to dental structures, because the erupted molars could be observed at a short imaging time (<1  min) and ICG dose almost did not affect imaging quality. Furthermore, OA-ICG-NIRF dental imaging could also identify the laser-treated abnormal erupted molars in both NIRF-I and NIRF-II. Overall, ICG-NIRF imaging could serve as a novel ionizing-radiation-free imaging method in first and second NIR windows. Mouthwash could help as a new non-invasive method for more efficient delivery of ICG dye to the dental structures for dental imaging at a short time and low ICG dose.

However, there are some limitations in this study. First, ICG-NIRF imaging is still 2D imaging. Second, although the laser ablation of dental follicles could cause the abnormal eruption of the molars, the animals used in the study did not have natural dental diseases. Third, ICG-NIRF imaging was only demonstrated in the rat model, and its feasibility needs to be further verified in other animal models, such as pig or rabbits. Finally, more experiments need to be done for the efficiency of ICG oral administration; for instance, how fast ICG would be absorbed and reach dental tissues through the blood circulation.
